# Molecular mechanism of extracellular matrix disorder in pelvic organ prolapses

**DOI:** 10.3892/mmr.2020.11721

**Published:** 2020-11-23

**Authors:** Liping Zhang, Fangfang Dai, Gantao Chen, Yanqing Wang, Shiyi Liu, Li Zhang, Shu Xian, Mengqin Yuan, Dongyong Yang, Yajing Zheng, Zhimin Deng, Yanxiang Cheng, Xiaofeng Yang

Mol Med Rep 22: 4611-4618, 2020; DOI: 10.3892/mmr.2020.11564

Subsequently to the publication of the above article, the authors have realized that the bar charts shown for Fig. 3A and B, as they appeared in the paper, were the same as the bar charts shown for Fig. 4B and D.

Fig. 3, as it should have appeared, is shown below. All the authors agree to this Corrigendum. Note that the revisions made to this figure do not adversely affect the results reported in the paper, or the conclusions stated therein. The authors regret that the duplication of the histograms in Fig. 4 as Fig. 3 was not noticed prior to the publication of this article, and offer their apologies to the Editor of *Molecular Medicine Reports* and to the readers of the Journal.

## Figures and Tables

**Figure 3.**
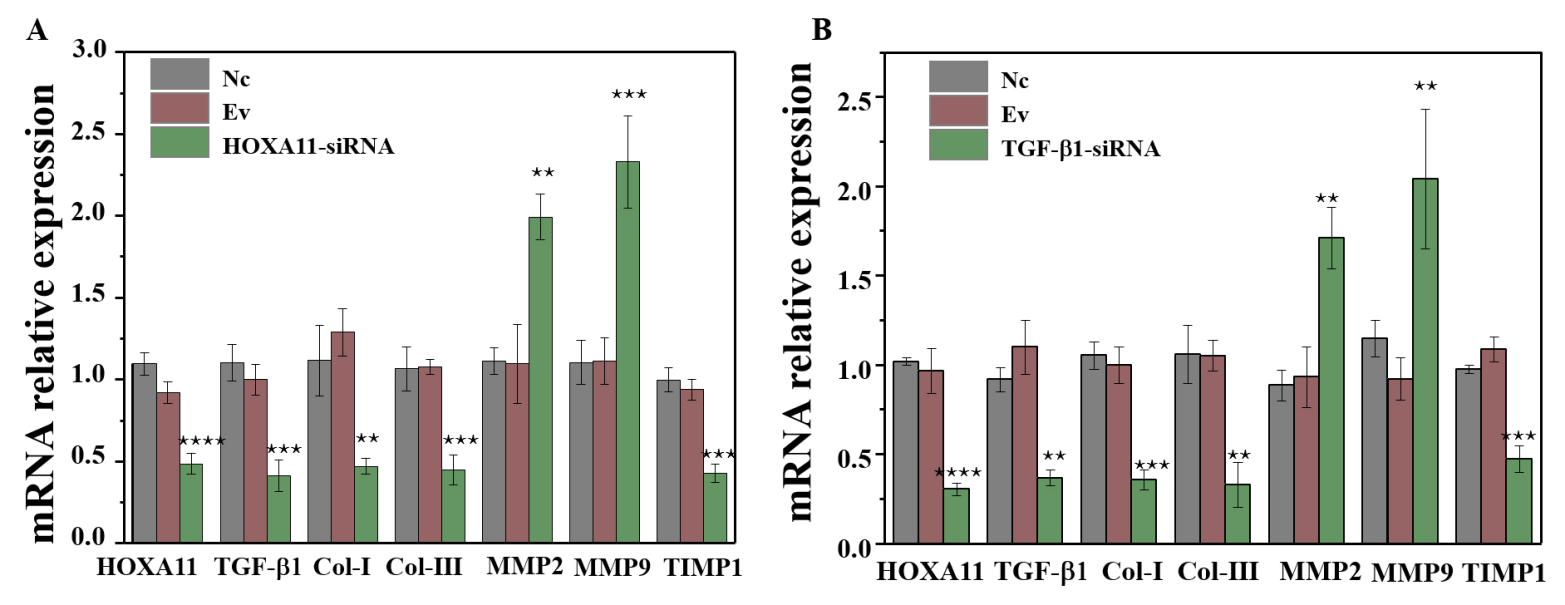
The molecular mRNA expression when HOXA11 or TGF-β1 knocked down. mRNA expression of Col-I, Col-III, HOXA11, TGF-β1, MMP-2, MMP-9 and TIMP1 in (A) HOXA11-siRNA group and (B) TGF-β1-siRNA group. *P<0.05, **P<0.01 and ***P<0.001 vs. siRNA-NC. Col, collagen; HOXA11, Homeobox11; TGF-β1, transforming growth factor β; MMP, matrix metalloproteinases; TIMP1, tissue metalloproteinase inhibitor 1; siRNA, small interfering RNA; NC, negative control.

